# Impacts of postpartum length at the initiation of the fixed-time artificial insemination protocol on pregnancy rates of *Bos indicus* beef cows

**DOI:** 10.1093/tas/txac095

**Published:** 2022-09-10

**Authors:** Ana Clara R Araújo, Reinaldo F Cooke, Izaias Claro Junior, Ocilon G Sá Filho, Carlos M S Borges, Paulo S L Sampaio, Benedito B Cocenza, Rodolfo S R Romero, José Henrique L M Tanner, José Luiz Moraes Vasconcelos

**Affiliations:** Faculdade de Medicina Veterinária e Zootecnia, Universidade Estadual Paulista, Botucatu, SP 18618-970, Brazil; Department of Animal Science, Texas A&M University, College Station, TX 77845, USA; Zoetis Animal Health – GERAR Group, São Paulo, SP 04583-110, Brazil; Zoetis Animal Health – GERAR Group, São Paulo, SP 04583-110, Brazil; Zoetis Animal Health – GERAR Group, São Paulo, SP 04583-110, Brazil; Zoetis Animal Health – GERAR Group, São Paulo, SP 04583-110, Brazil; Zoetis Animal Health – GERAR Group, São Paulo, SP 04583-110, Brazil; Zoetis Animal Health – GERAR Group, São Paulo, SP 04583-110, Brazil; Zoetis Animal Health – GERAR Group, São Paulo, SP 04583-110, Brazil; Zoetis Animal Health – GERAR Group, São Paulo, SP 04583-110, Brazil

**Keywords:** *Bos indicus*, cows, days postpartum, fixed-time artificial insemination, pregnancy

## Abstract

The shortest interval between calving and initiation of fixed-time artificial insemination (FTAI) protocols recommended in Brazilian cow–calf systems is 30 d, based on research that characterized uterine involution and incidence of uterine disorders in *Bos taurus* females. Prevalence of uterine disorders such as subclinical endometritis is limited in Nelore (*B. indicus*) cows as early as 28 d after calving. We hypothesized that Nelore cows can receive an FTAI protocol as early as 20 d postpartum (DPP) and still experience satisfactory reproductive results. This study evaluated pregnancy rates in 5,258 Nelore cows (*n* = 1,703 primiparous and 3,555 multiparous) according to DPP at the initiation of the FTAI protocol. Cow body condition score (BCS) was recorded at FTAI, and pregnancy diagnosis was performed ~30 d after FTAI. Cows were ranked within parity by DPP at the initiation of the FTAI protocol and classified according to 5-d intervals (e.g., ≤15 DPP, 16 to 20 DPP, 21 to 26 DPP, until cows with ≥76 DPP). Data were analyzed within parity, using cow as experimental unit and orthogonal polynomial contrasts (linear, quadratic, or cubic) generated using the mean DPP of each DPP class. In both parities, cow BCS at FTAI decreased linearly (*P* ≤ 0.01) with the advance of DPP (e.g., 4.79, 4.00, and 3.73 in primiparous, and 4.95, 3.70, and 3.23 in multiparous cows classified as ≤15 DPP, 36 to 40 DPP, ≥76 DPP, respectively). The pregnancy rate to FTAI was affected quadratically (*P* < 0.01) by DPP for both parities. In primiparous cows, the pregnancy rate increased until 36 to 40 DPP (60%), remained near this level until 51 to 60 DPP, and then decreased with the advance of DPP, whereas cows classified as 21 to 25 DPP expressed satisfactory results (41.5%). In multiparous cows, the pregnancy rate increased until 46 to 50 DPP (70.8%), remained near this level until 56 to 60 DPP, and then decreased with the advance of DPP, whereas cows classified as 21 to 25 DPP also expressed satisfactory results (63.6%). Collectively, primiparous and multiparous Nelore cows evaluated herein experienced optimal pregnancy rates when the FTAI protocol was initiated within 30 to 60 DPP, although reasonable outcomes were observed when the FTAI protocol was initiated as early as 21 DPP. Hence, the interval between calving and initiation of the FTAI protocol can be shortened by 10 d in Nelore females and still yield acceptable pregnancy rates, which can be of great value to cows that calve immediately prior to or during the annual breeding season.

## INTRODUCTION

Reproductive efficiency of beef females is critical for the productivity of cow–calf operations (Lamb et al., 2008) and has direct implications for beef supply by determining the number of cattle available for harvest (Cooke et al., 2020). Given the continuing increase in worldwide beef demand (FAO, 2009), management strategies that enhance cattle reproduction are warranted to ensure the sustainability of cow–calf systems and address the global need for animal protein. The use of fixed-time artificial insemination (FTAI) improved reproductive and overall beef production efficiency in countries that lead in world beef production, particularly Brazil (Vasconcelos et al., 2014; FAOSTAT, 2019). More than 20% of females in Brazil cow–calf systems are inseminated annually (ASBIA, 2021), where the use of FTAI protocols tailored for *Bos indicus* cattle consistently yields pregnancy rates above 50% (Vasconcelos et al., 2014).

The shortest interval between calving and initiation of FTAI protocols recommended for cow–calf systems in Brazil is 30 d (Vasconcelos et al., 2014), although this concept was mostly derived from research with *Bos taurus* females including Holstein cows (Crowe et al., 2014). Insufficient nutritional status, calf sucking, and time required for uterine involution directly contribute to postpartum anestrous (Lamb et al., 1999; Ungerfeld et al., 2011; Crowe et al., 2014). The first two factors can be mitigated, at least partially, with the use of exogenous hormones included in FTAI protocols in Brazil such as equine chorionic gonadotropin (eCG), progesterone, and estradiol (Baruselli et al., 2004; Vasconcelos et al., 2014). Uterine involution is regulated by inherent and environmental factors, particularly endometritis that can affect up to 70% of postpartum dairy cows (LeBlanc, 2008). Nonetheless, postpartum uterine diseases are typically considered uncommon or with limited reproductive consequences for beef cows (Santos et al., 2009).

Pfeifer et al. (2018) and Oliveira Filho et al. (2022) recently reported limited prevalence of endometritis in *B. indicus* cows as early as 20 d postpartum (DPP), particularly when compared with the incidence of such disorder in dairy cattle (LeBlanc, 2008). Based on this rationale, we hypothesized that *B. indicus* cows can receive FTAI protocols based on progesterone, estradiol, and eCG prior to as early as 20 DPP and still experience satisfactory reproductive results. Despite the massive body of research studies that developed FTAI protocols for *B. indicus* cows (Baruselli et al., 2017; Vasconcelos et al., 2017; Cooke et al., 2020), limited studies were specifically designed to assess pregnancy rates to these protocols according to postpartum length. Hence, this experiment addressed this gap in knowledge and tested our hypothesis by comparing pregnancy rates in *B. indicus* cows according to their DPP at the initiation of an FTAI protocol commonly adopted by Brazilian cow–calf systems (Vasconcelos et al., 2017).

## MATERIALS AND METHODS

This experiment was conducted from October 2020 to March 2021 in six commercial cow–calf ranches located in central Brazil. All animals utilized herein were cared for in accordance with the practices outlined in the *Guide for the Care and Use of Agricultural Animals in Agricultural Research and Teaching* (FASS, 2020).

### Animal Management

A total of 5,258 suckled, nonpregnant Nelore cows were assigned to the experiment (*n* = 1,703 primiparous and 3,555 multiparous). Cows were managed according to the general management scheme of each operation, in groups maintained in individual *Brachiaria* spp. pastures according to parity with an average of 82 cows each (range = 30 to 200 cows/group; ranch A = 14 groups, ranch B = 9 groups; ranch C = 16 groups; ranch D = 10 groups; ranch E = 9 groups; and ranch F = 7 groups).

Reproductive management also followed the management scheme of these operations, and all groups were assigned to the FTAI protocol (day 0 to 11) described by Peres et al. (2009). Cows received a 2 mg injection (i.m.) of estradiol benzoate (Gonadiol; Zoetis, São Paulo, SP, Brazil) and an intravaginal progesterone-releasing device (CIDR, containing 1.9 g of progesterone; Zoetis) with no previous use on day 0, a 12.5 mg injection (i.m.) of PGF_2α_ (Lutalyse; Zoetis) on day 7, followed by CIDR removal in addition to 0.6 mg injection (i.m.) of estradiol cypionate (ECP; Zoetis) and 300 IU injection (i.m.) of eCG (Novormon; Zoetis) on day 9. Cows were assigned to FTAI on day 11 by one of three technicians within each group, using semen from multiple sires.

For the purposes of this experiment, cows were ranked by parity and DPP at initiation of the FTAI protocol (day 0) and grouped according to 5-d intervals (Tables 1 and 2) but in a manner that each class had a representative number of animals. Due to limited number of cows, those with DPP ≤ 15, between 61 and 75 DPP, and DPP ≥ 76 were grouped together in both parities. Moreover, primiparous cows were grouped as 51 to 60 DPP because only 11 cows had a DPP between 56 and 60 d (Table 1). The range in DPP values observed was 4 to 95 d in primiparous, and 3 to 93 d for multiparous cows. Cow body condition score (BCS; Wagner et al., 1988; 1 to 9 scale) was recorded at the time of FTAI, and pregnancy status was verified by detecting a viable conceptus with transrectal ultrasonography (5.0-MHz transducer; 500V, Aloka, Wallingford, CT) 28 to 35 d after FTAI.

**Table 1.** Number and BCS (Wagner et al., 1988) of primiparous Nelore (*Bos indicus*) cows according to the classification of DPP at initiation of a fixed-time artificial insemination protocol (Peres et al., 2009)^†^

Item	*n*	Mean DPP	BCS
≤15	41	11.4	4.79
16 to 20	186	17.3	4.46
21 to 25	178	22.9	4.11
26 to 30	192	27.7	4.26
31 to 35	224	32.5	4.12
36 to 40	188	37.4	4.00
41 to 45	175	42.5	4.06
46 to 50	181	46.7	3.77
51 to 60	107	52.3	3.71
61 to 75	82	69.0	3.69
≥76	149	82.0	3.73
SEM	—	0.52	0.004
Linear contrast	—	<0.01	0.02
Quadratic contrast	—	0.99	0.82
Cubic contrast	—	0.99	0.33

Orthogonal polynomial contrasts were tested to determine the impacts of DPP at the initiation of FTAI on BCS. Contrast coefficients were generated using the mean DPP of each DPP class.

**Table 2.** Number and BCS (Wagner et al., 1988) of multiparous Nelore (*Bos indicus*) cows according to the classification of DPP at initiation of a fixed-time artificial insemination protocol (Peres et al., 2009)^†^

Item	*n*	Days postpartum	BCS
≤15	186	12.6	4.95
16 to 20	410	18.0	4.89
21 to 25	188	23.7	4.50
26 to 30	247	27.9	4.55
31 to 35	409	32.7	3.79
36 to 40	317	38.6	3.70
41 to 45	375	43.8	3.63
46 to 50	374	48.3	3.25
51 to 55	409	53.1	3.65
56 to 60	271	58.7	3.27
61 to 75	232	67.5	3.45
≥76	137	83.8	3.23
SEM	—	0.31	0.007
Linear contrast	—	<0.01	<0.01
Quadratic contrast	—	0.99	0.02
Cubic contrast	—	0.99	0.56

Orthogonal polynomial contrasts were tested to determine the impacts of DPP at the initiation of FTAI on BCS. Contrast coefficients were generated using the mean DPP of each DPP class.

### Statistical Analyses

Data were analyzed within each parity using cow as experimental unit, and Satterthwaite approximation to determine the denominator degrees of freedom for the tests of fixed effects. For all analyses, significance was set at *P* ≤ 0.05, and tendencies were determined if *P* > 0.05 and *P* ≤ 0.10. Quantitative data were analyzed with the MIXED procedure of SAS (SAS Inst., Inc., Cary, NC), and binary data were analyzed with the GLIMMIX procedure of SAS (SAS Inst., Inc) with a binomial distribution and logit link function. All model statements contained the effect of DPP class, with ranch and group(ranch) as random variables. Sire and inseminator were also included as random variables for the analysis of pregnancy rates to FTAI. All results are reported as least square means, and orthogonal polynomial contrasts were tested to determine the impacts of DPP at the initiation of FTAI on BCS and pregnancy rates (linear, quadratic, or cubic). Contrast coefficients were generated using the mean DPP of each DPP class (Tables 1 and 2) with the IML procedure of SAS (SAS Inst. Inc.). The contrasts described above were chosen given their relevance to our hypothesis, whereas quartic and higher-degree contrasts often have limited biological meaning (Kaps and Lamberson. 2017). If multiple contrasts were significant (*P* ≤ 0.05), the contrast with the greatest *F*-value is discussed. The probability of cows becoming pregnant was also evaluated according to DPP at the initiation of the FTAI protocol. The GLM procedure of SAS was used to determine if DPP influenced reproductive responses linearly, quadratically, or cubically. The LOGISTIC procedure was used to generate the regression model, determine the intercept and slope(s) values according to maximum likelihood estimates from each significant continuous order effect, and the probability of pregnancy was determined according to the following equation: Probability = (e^logistic equation^)/(1 + e^logistic equation^). Logistic curves were constructed with all DPP values recorded for each parity during the experiment.

## RESULTS AND DISCUSSION

Across ranches, the mean DPP at the initiation of the FTAI protocol was 40.5 ± 0.3 d (39.7 ± 0.4 d for primiparous and 40.8 ± 0.4 d for multiparous cows), and the mean BCS at FTAI was 4.03 ± 0.01 (4.09 ± 0.03 for primiparous and 4.00 ± 0.01 for multiparous cows). Mean pregnancy rate to FTAI was 57.1 ± 0.7% (3,002 pregnant cows/5,258 total cows), being 48.9 ± 1.2% for primiparous (832 pregnant cows/1,703 total cows) and 61.0 ± 0.8% for multiparous cows (2,170 pregnant cows/3,555 total cows). Overall, these values are typical of cowherds in Brazil that adopt FTAI (Baruselli et al., 2017; Vasconcelos et al., 2017; Cooke et al., 2020), corroborating the relevance of this experiment to commercial cow–calf systems based on *B. indicus* females in South America and other tropical regions of the world.

As designed, mean DPP was affected linearly (*P* < 0.01) by the DPP class for primiparous and multiparous cows (Tables 1 and 2), and this was analysis performed to generate least square means and subsequent contrast coefficients. Cow BCS at FTAI decreased linearly (*P* ≤ 0.02) according to DPP for both parities (Tables 1 and 2), which was resultant of BCS mobilization to support increased milk production as DPP advanced (Alderton et al., 2000; Mulliniks et al., 2012; Ayres et al., 2014). The negative relationship between DPP and cow BCS, even after cows reach their lactation peak, is typical of cow-calf systems based on low-quality tropical forages without or limited supplementation programs (Vasconcelos et al., 2014; Cooke et al., 2020). For this reason, Meneghetti and Vasconcelos (2008) suggested that FTAI protocols should start as soon as 30 DPP to alleviate or prevent excessive BCS loss, particularly in young cows with increased nutritional demands compared with mature cows (NASEM, 2016). Nonetheless, the negative association between DPP and BCS was also noted and should not be overlooked for multiparous cows, given the established effects of BCS on the reproductive function of beef females (Kunkle et al., 1994; Hess et al., 2005; Cooke et al., 2021).

**Figure 1.**
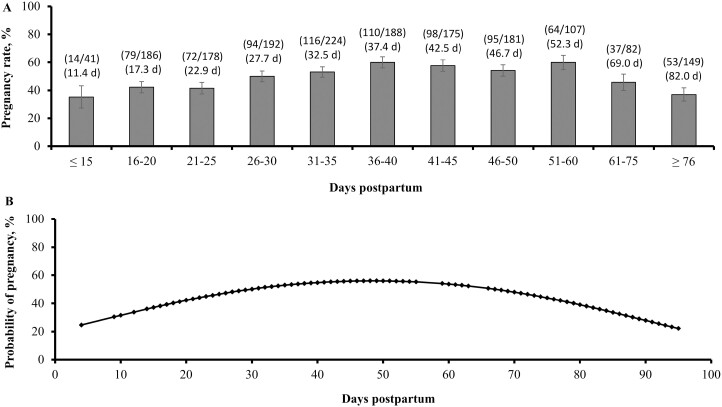
Pregnancy rates (A) and probability of pregnancy (B) in primiparous Nelore (*Bos indicus*) cows according to the classification of DPP at initiation of a fixed-time artificial insemination protocol (Peres et al., 2009). Quadratic effects were detected (*P* < 0.01) for both analyses. Within parenthesis in (A), the top values represent pregnant cows divided by total cows, and the bottom values represent the mean DPP of each class used to generate contrast coefficients.

**Figure 2.**
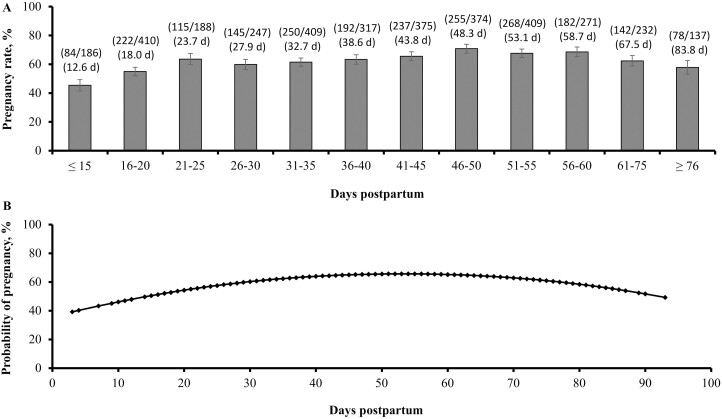
Pregnancy rates (A) and probability of pregnancy (B) in multiparous Nelore (*Bos indicus*) cows according to the classification of DPP at initiation of a fixed-time artificial insemination protocol (Peres et al., 2009). Quadratic effects were detected (*P* < 0.01) for both analyses. Within parenthesis in (A), the top values represent pregnant cows divided by total cows, and the bottom values represent the mean DPP of each class used to generate contrast coefficients.

The pregnancy rate to FTAI was affected by DPP quadratically (*P* < 0.01) for both parities. In primiparous cows, pregnancy rates increased until 36 to 40 DPP at ~60%, remained near this level until 51 to 60 DPP, and then decreased with the advance of DPP (Figure 1a). In multiparous cows, pregnancy rates increased until 46 to 50 DPP at ~70%, remained near this level until 56 to 60 DPP, and then decreased with the advance of DPP (Figure 2a). The probability of pregnancy according to DPP was also affected quadratically in both parities, yielding maximum values at 48 DPP in primiparous cows (Figure 1b) and 54 DPP in multiparous cows (Figure 2b). Together, these results corroborate that *B. indicus* females experience optimal pregnancy rates to FTAI when the protocol utilized herein is initiated shortly after 30 DPP in primiparous and 50 DPP in multiparous cows. The decrease in pregnancy rates to FTAI in cows with elevated DPP (≥ 61 DPP) can be associated, at least partially, with the concurrent reduction in BCS to values known to impair cow reproductive function (Cooke et al., 2021). This latter outcome highlights the importance of adequate nutritional management of postpartum beef cows, particularly those that calve early in the calving season to prevent excessive BCS loss before the next breeding season (Hess et al., 2005).

The main goal of this experiment, however, was to characterize pregnancy rates in cows that initiate the FTAI protocol around 20 DPP. Pregnancy rates to FTAI in primiparous cows classified as 21 to 25 DPP were 41.5%, a 1.4-fold decrease in comparison with values noted for primiparous cows classified as 36 to 40 DPP (60.0%; Figure 1a). In multiparous cows, pregnancy rates to FTAI were 63.6% at 21 to 25 DPP, representing a 1.1-fold reduction when compared with cows classified as 46 to 50 DPP (70.8%; Figure 2a). Despite the considerable reduction compared with DPP classes that yielded the greatest values for pregnancy rates, initiating the FTAI protocol as early as 21 DPP yielded satisfactory results according to the recent averages reported for primiparous and multiparous *B. indicus* cows in Brazil (Baruselli et al., 2017; Vasconcelos et al., 2017), including cows assigned to the FTAI protocol between 30 and 40 DPP (Peres et al., 2009; Cooke et al., 2020; Carvalho et al., 2022). To our knowledge, these results are novel and should be associated with the greater BCS of cows at early DPP, the use of exogenous hormones that compensate for nutritional deficiencies and calf suckling, and limited incidence of postpartum uterine diseases in beef females (Baruselli et al., 2004; Meneghetti and Vasconcelos, 2008; Santos et al., 2009). More specifically, BCS was greater at early DPP in both primiparous and multiparous cows, whereas the biological reasons and reproductive impacts of this outcome were already discussed herein (Meneghetti and Vasconcelos, 2008; Cooke et al., 2021). The use of eCG in FTAI protocols alleviates the negative effects of calf suckling on LH secretion and follicular development in postpartum beef cows (Kuran et al., 1996; Peres et al., 2009). Both Pfeifer et al. (2018) and Oliveira Filho et al. (2022) reported a negative association between subclinical endometritis, based on the proportion of polymorphonuclear cells in uterine samples and pregnancy rates of FTAI in Nelore cows. Nonetheless, Oliveira Filho et al. (2022) reported that only 6% of cows had proportion of polymorphonuclear cells ≥ 5% between 28 and 40 d after calving, which is a threshold typically used to diagnose endometritis in dairy cattle (Madoz et al., 2013; Wagener et al., 2017). Hence, delayed uterine involution due to postpartum endometritis appears to have limited prevalence and reproductive consequences for Nelore cowherds (Santos et al., 2009).

Collectively, primiparous and multiparous Nelore cows evaluated in this experiment expressed optimal pregnancy rates when the FTAI protocol was initiated between 30 and 60 DPP. Reasonable results, however, were noted when the FTAI protocol was initiated as early as 21 DPP in both parities. Therefore, the interval between calving and initiation of the FTAI protocol can be shortened by 10 d and still yield acceptable pregnancy rates in Nelore females. This strategy is of great value to cows that calve immediately prior to or during the annual breeding season, increasing their chance to conceive and contribute to reproductive and overall efficiency of cow–calf systems (Lamb et al, 2008; Cooke et al., 2021).
